# Strategies for rural areas: The development of and initial experiences with a training course for physicians from third countries to prepare them for medical practice in Germany

**DOI:** 10.3205/zma001233

**Published:** 2019-05-16

**Authors:** Karolin Hahn, Jost Steinhäuser

**Affiliations:** 1 Universitätsklinikum Schleswig-Holstein, Campus Lübeck, Institut für Allgemeinmedizin, Lübeck, Germany

**Keywords:** Physicians from non-EU member states, third-country physicians, curriculum, recognition of qualifications, theoretical exam, preparatory course

## Abstract

**Aim: **In Germany there is an increasing shortage of physicians, especially in rural areas. Strategies that make use of medical doctors from non-EU member states could effectively counteract this problem more rapidly than other strategies, including those that focus on education. Physicians from third countries must first demonstrate evidence of their professional qualifications on an exam. The failure rate in Schleswig-Holstein is approximately 50%.

The specific aim of the 80-hour training course is to prepare third-country physicians for the practice of medicine in Germany and to provide exposure to the rural setting, regardless of whether or not these physicians have already taken an exam to receive formal recognition of their professional qualifications.

**Method:** The need for post-licensure training courses was discerned in interviews with third-country physicians and examiners. The course was also evaluated using different instruments and then revised accordingly.

**Results: **The training program has been held four times with a total of 52 third-country physicians; the program was given the very good rating of 1.4 on the traditional German academic grading scale. In addition to the 10-day training course, the participants had access to an online medical learning platform. Moreover, information on working in rural setting and a field trip to a variety of medical institutions in a rural region was integrated into the course. The majority of the participants used the course as additional preparation for the exam. Their willingness to later practice medicine in a rural setting was high with 89% of participants.

**Conclusion: **The evaluation results suggest that such an intensive training program is suitable to prepare third-country physicians for medical practice in Germany and in particular in rural regions.

## Zusammenfassung

**Zielsetzung: **In Deutschland existiert ein zunehmender Ärztebedarf, vor allem in ländlichen Regionen. Strategien dagegen, die auf der Ebene von Ärzten aus Drittstaaten (ÄaD) ansetzen, können rascher als solche, die beispielsweise in der Ausbildung ansetzen, Wirkung zu zeigen. ÄaD müssen zunächst durch eine Kenntnisprüfung ihre Eignung unter Beweis stellen. Die Nichtbestehensquote liegt in Schleswig-Holstein bei etwa 50%.

Ziel des eigens entwickelten 80 Stunden Kurses war es, unabhängig davon, ob ÄaD eine Kenntnisprüfung bereits abgelegt haben, gezielt auf die ärztliche Tätigkeit in Deutschland vorzubereiten und dabei insbesondere den ländlichen Raum näher zu bringen.

**Methodik: **Die Bedarfe für den Fortbildungskurs wurden mittels Interviews mit Prüfern von Kenntnisprüfungen und ÄaD erhoben. Der Kurs wurde zudem mit verschiedenen Instrumenten evaluiert und daraufhin überarbeitet.

**Ergebnisse:** Der Kurs wurde vier Mal mit insgesamt 52 ÄaD durchgeführt und wurde nach Schulnoten mit 1,4 evaluiert. Neben dem zehntägigen Fortbildungsangebot wurde den Teilnehmern (TN) der Zugang zu einer onlinebasierten medizinischen Lernplattform angeboten. Weiterhin wurden Inputs zum Arbeiten im ländlichen Raum sowie ein Ausflug in den ländlichen Raum mit dem Besuch unterschiedlichster medizinischer Einrichtungen in den Kurs integriert. Die Mehrheit der TN nutzte den Kurs als zusätzliche Vorbereitung auf die Kenntnisprüfung. Die Bereitschaft, später im ländlichen Raum zu praktizieren, war mit 89% der TN hoch.

**Schlussfolgerung: **Die Evaluationsergebnisse legen nahe, dass ein solcher Intensivkurs geeignet ist, ÄaD auf ihre ärztliche Tätigkeit in Deutschland und insbesondere in ländlichen Regionen vorzubereiten.

## 1. Introduction

### 1.1. Background on physicians from third countries

Although Germany has a comparatively high density of medical doctors with 41 physicians per 10,000 inhabitants and the number of physicians is steadily increasing, there is the threat of geographical shortages [1], [2]. Different strategies are needed to counteract this shortage of physicians. However, strategies focusing on education require around 11 years before they begin to show an effect. Strategies that focus on physicians from third countries could show more rapid effects in terms of providing healthcare, since these physicians have already been licensed and may even have a medical specialty. Currently, 11% of medical doctors do not have German citizenship [1]. Every third person in Germany has an immigrant background [3].

Formal recognition of professional qualifications is only possible in Germany if an equivalent level of education can be demonstrated. For physicians from EU member states this recognition takes the form of medical licensure (Approbation); in contrast, physicians from third countries may only apply for professional licensure under Section 10 of the regulations governing medical doctors (Bundesärzteordnung) if they can provide evidence of a job offer. An exam must be taken and passed to receive such recognition; the rules dictating how this is done are applied differently in each German state [3]. The exams cover material on internal medicine and surgery; there are no national standards. All third-country physicians have the right to receive an appointment to take the exam within six months of application [https://www.gesetze-im-internet.de/_appro_2002/__37.html].

Since the recognition process is handled differently in each German state, there are no comparative national statistics on third-country physicians. It is impossible to say how many third-country physicians are waiting to take an exam or how many go through the recognition procedure each year. However, it is known that up to two-thirds of immigrants with university degrees do not receive recognition of their qualifications in Germany [4].

The exam administered in Schleswig-Holstein also contains questions on general practice, a physical examination of an inpatient and documentation of the related epicrisis. The failure rate for the exam in Schleswig-Holstein is around 50% [5].

There are substantial discrepancies among the applicants regarding prior knowledge making compensatory measures recommendable to lower the failure rate [6]. Furthermore, it is also known that foreign doctors in Germany need to adjust to fulfill different functions, for instance, the drawing of blood as a medical task [7].

In Germany, however, there has been a lack of targeted interventions to prepare third-country physicians for medical practice in rural German areas. On one hand, exposure to rural regions is critical in order to realistically imagine working in such a setting; and on the other, broad medical expertise is a key requirement when actually taking up rural medical practice [8].

#### 1.2. Aim

The aim of this report is to present the development of a post-licensure training program for physicians from non-EU countries. The program was specifically designed as preparation for medical practice in the rural regions of the German state Schleswig-Holstein. This training program was designed without regard for type of medical licensure or specialty.

## 2. Method

### 2.1. Design of the post-licensure training program

When developing and designing this post-licensure course, it was possible to apply previous experience in developing curricular training courses, the competency-based curriculum in general practice, and professional reentry seminars [9], [10], [11].

Based on Kern et al. [12], the perspectives of third-country physicians who had already taken an exam to receive professional recognition or who were poised to take such an exam were included in the development of the training course, as were the perspectives of experienced examiners in Schleswig-Holstein. These perspectives were gathered through telephone interviews that were conducted according to guidelines and from a survey of a focus group [Guidelines, see attachment 1 ]. The interviews were recorded using a digital recording device and then transcribed.

#### 2.2. Recruiting interviewees

Participant recruitment for the interviews was done through the Schleswig-Holstein Medical Association (Ärztekammer Schleswig-Holstein) for the examiners and through the State Medical Examination Board (Landesprüfungsamt Schleswig-Holstein) and the network called Integration durch Qualifizierung (IQ) [http://www.iq-netzwerk-sh.de/] for the third-country physicians.

#### 2.3. Recruiting participants for the training program

In addition to having a medical degree awarded by a non-EU member state, the expected minimum level of German language proficiency was B2. The course was open to all third-country physicians regardless of their (desired) specialty.

Information on the training program was presented on the website of Schleswig-Holstein’s academy for post-licensure training and post-graduate education (Akademie für medizinische Fort- und Weiterbildung der Ärztekammer Schleswig-Holstein) [https://www.aeksh.de/fortbildung] and shared on the IQ network. In addition, information was published on the educational portal of the Federal Employment Agency in Germany [13].

#### 2.4. Evaluation of the training program

According to the evaluation model proposed by Kirkpatrick to evaluate training courses, evaluation ideally takes place on four levels: reaction, learning, behavior, and results [14]. Participation in the evaluation survey was voluntary and anonymous.

First, at the beginning of the course participants were asked to do a self-assessment using a “medical practitioner barometer”. This questionnaire asked about the CanMeds competencies in the form of a self-assessment [15]. The scale used on the questionnaire was a five-point Likert scale ranging from 1=“agree completely” to 5=“disagree completely”.

The first level of Kirkpatrick’s model (reaction) was measured by a daily evaluation [16] of the course sessions and an overall evaluation. On the daily evaluations, questions were asked about course content, presentation (teaching, etc.), opportunities to participate, work atmosphere, and practical relevance of the individual course blocks and instructors. The daily evaluation contained a six-point Likert scale ranging from 1=“very satisfied” to 6=“very dissatisfied”. To arrive at the overall evaluation, the mean value was calculated based on all questionnaires.

The subjective growth in knowledge was measured as of the second training course and focused on the second level: learning. The questions were answered using a five-point Likert scale with the options: 1=”no knowledge gain” to 5=”extreme knowledge gain”.

After each training course, participant feedback, both spoken and written, was used to make adjustments to the program.

#### 2.5. Evaluation of the field trip

An evaluation with a pre-post design was used to ascertain the possible effect of exposure to the rural setting (“gangway”) on participants’ attitudes toward working in rural areas [17]. Before and after the field trip, participants responded to the question: What is the probability that you will practice in a rural area in the future? This survey was also voluntary and anonymous. The evaluation of the excursion, as with the evaluation of the training course itself, took satisfaction (level 1: reaction) and potential learning as a result of the experience (level 2: learning) into account.

#### 2.6. Process evaluation

The participants were contacted by telephone several months after completing the course, at least once a year, and asked about their professional experience and development. Levels 3 and 4 (behavior, results) were thus also taken into consideration.

#### 2.7. Analysis

The content of the interviews was analyzed independently by both authors (KH, health science and JS, medicine). They applied qualitative content analysis according to Mayring [18]. A discussion then followed regarding the identified themes and continued until consensus was reached.

The descriptive analysis of the evaluation questionnaires was done using IBM SPSS Statistics, version 24.0 (IBM Corp., Armonk, NY).

#### 2.8. Ethics

Approval from the Ethics Commission of Lübeck University was received for the design of the post-licensure training program (file no.16-314).

## 3. Results

### 3.1. Interviews

A total of seven interviews were conducted with examiners, who were on average 58 years old and without exception male.

The third-country physicians were surveyed in individual interviews and in a focus group. Overall, it was possible to include the points of view of nine third-country physicians. These physicians were on average 30 years old and 18% were female.

The main categories with the corresponding codes and examples of statements made by examiners and third-country physicians are presented in attachment 2 .

All in all, the project team received a clear sense of the potential for improvements to the theoretical exams and to the preparation for medical practice in Germany. All of the surveyed examiners were in agreement that the examinees demonstrated highly heterogeneous knowledge and that occasionally knowledge classified as basic was lacking. This was explained by the nature of medical study in some countries of origin (e.g. very few practical elements). Further causes were presumed to be the German working conditions and lack of opportunity for rotation, as well as previous experience that was too specialized.

In the interviews it was also stated that time to study for the exam was often lacking, especially if the participants had already taken up medical practice on the basis of a temporary medical license.

Lastly, relevant topics were identified that should be fixed components in a training program for third-country physicians. These ranged from basic knowledge in the exam subjects of surgery, internal medicine, general practice, and pharmacology to the structures within the German healthcare system.

#### 3.2. Course sequence

The post-licensure training program involves a 10-day course with a total of 80 hours and has been held four times as part of the larger project to qualify foreign medical doctors for rural practice in northern Germany (LandärztInnen Nord – Anpassungsqualifizierung für ausländische Ärztinnen und Ärzte). The training courses were conducted with the support of the Schleswig-Holstein Medical Association’s academy dedicated to post-licensure training and post-graduate education in medicine [https://www.aeksh.de/fortbildung].

Each day was divided into four blocks, two in the morning and two in the afternoon. The morning blocks consisted mostly of interactive input. Afternoons were reserved primarily for practical units followed by time during which the course attendees could engage in targeted self-guided study and review or expand on the learned material under competent tutelage.

The first day began with a survey of the attendees’ expectations and individual strengths which led to an initial discussion about what the attendees wished to focus on. On the last day a final discussion was held to ascertain if the expectations had been fulfilled.

The topics covered by the training course ranged from insights into the German healthcare system, the responsibilities of public health authorities, general practice, internal medicine, surgery and emergency medicine, all the way to topics such as polypharmacy, doctor/patient communication, clinical examinations and how to apply guidelines. This covered all of the topics mentioned in the interviews.

The instructors were specialized in the particular subjects they taught and were selected for the academy on the basis of previous positive teacher ratings, thus making it possible to draw on a very wide range of experience.

The use of simulated patients allowed attendees to not only refresh their knowledge of general examination techniques, but also to record structured case histories in German. Phantoms, e.g. to practice resuscitation, were also utilized, as were learning materials to impart information on surgical suturing techniques.

The topics “dealing with finding time to study” and “addressing heterogeneous standards of knowledge” were covered with the help of an online medical learning program. The program AMBOSS offered by MIAMED [https://www.miamed.de/] was used for this. Attendees had the opportunity each day to repeat the topics covered in the training sessions and to expand their own self-guided study. Physicians with knowledge of the program were present as instructors in the block sessions to adequately answer both medical questions and those concerning the learning platform.

**Figure 1:**
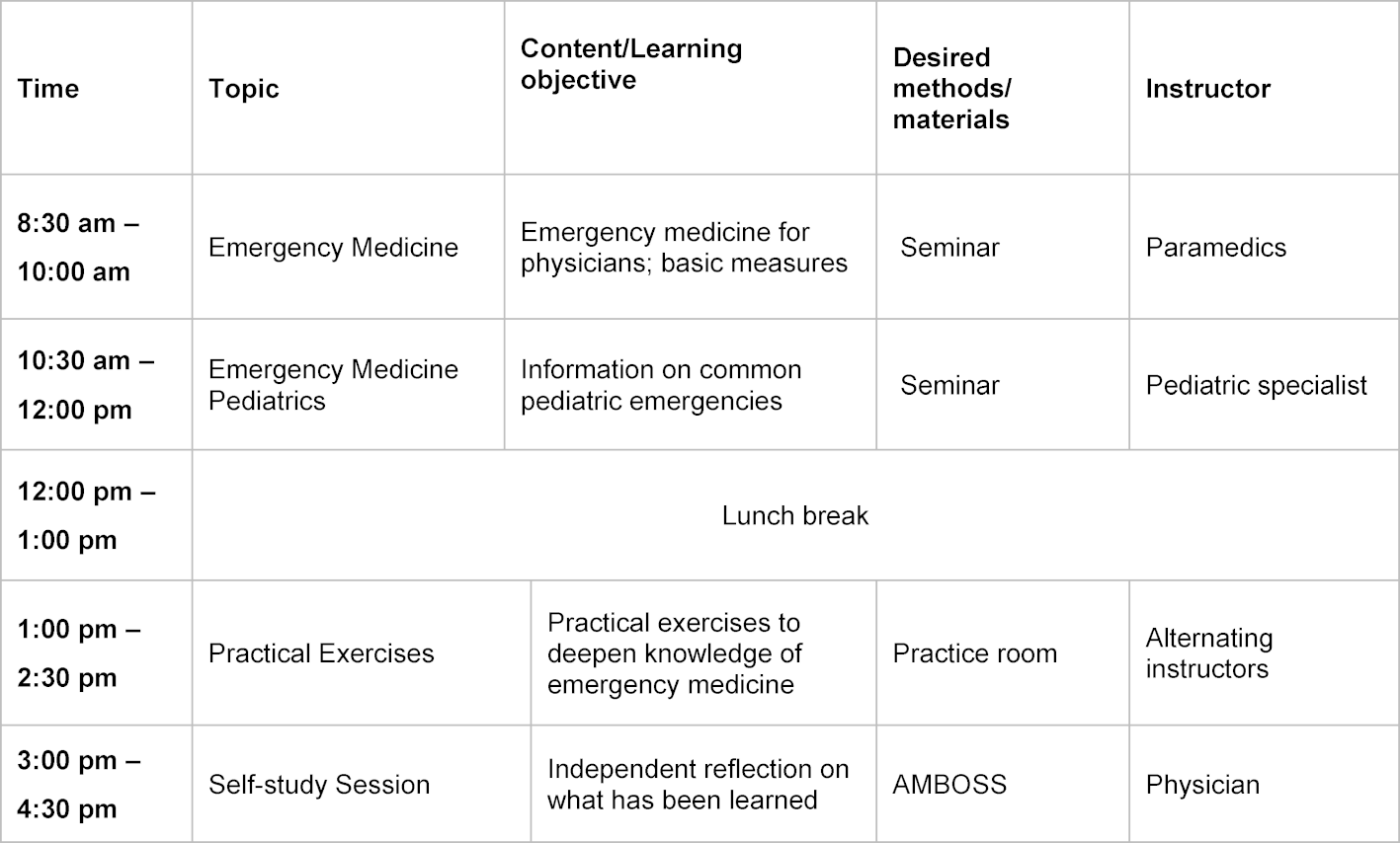
Overview presenting the schedule of a day in the training course

An example schedule for a day in the post-licensure training course is presented in figure 1 (Fig. 1).

#### 3.3. Sociodemographic profile of the attendees

A total of 52 third-country physicians have attended four separate training courses. Syria was the country of origin most often represented with 26 attendees; three attendees came from the Ukraine, another three from Egypt; two attendees were from Jordan, another two from Poland, and one attendee each came from Afghanistan, Azerbaijan, Algeria, Bolivia, Chile, the Dominican Republic, Greece, India, Columbia, Mexico, Russia, Saudi Arabia, Serbia, Turkey and Venezuela. The average age was 34.9 years. Three-fourths of the attendees were male (75%).

#### 3.4. Evaluation

The four courses were given an overall rating of 1.42 on a six-point Likert scale of 1=“very satisfied” to 6=“very dissatisfied.”

Details regarding the self-assessments using the “medical practitioner barometer” are contained in table 1 (Tab. 1). Overall, the attendees rated themselves as good. The items relevant to giving and receiving feedback were rated the lowest. The attendees assessed their abilities as being particularly good in regard to protecting patients from receiving too much medical treatment and including them in the process of making medical decisions.

The results of the self-assessments are shown in table 1 (Tab. 1).

##### 3.4.1. Daily evaluations

Table 2 (Tab. 2) provides an overview of the results gathered from the daily evaluations. The ratings given in the individual categories improved from one course to the next.

##### 3.4.2. Growth in knowledge

Starting with the second time the training course was offered, attendees were requested to assess their subjective knowledge gain on a five-point Likert scale with the response options of 1=“no knowledge gain” to 5=“extreme knowledge gain.” In all of the courses, the attendees reported that they felt the greatest knowledge increase in the subject of general practice, followed by working with guidelines and emergency medicine. A moderate gain in knowledge was registered by the attendees in pharmacology.

The overall results can be found in table 3 (Tab. 3).

##### 3.4.3. Rural field trip “gangway”

So that attendees could gain insights into working in rural areas, information was given in lectures and presentations and in the form of a field trip. This all-day excursion included visits to different rural medical facilities and discussions with representatives from local government. The time traveling between medical facilities was used to present information on the rural setting and other healthcare topics.

The field trip has taken place and been evaluated three times already. It was piloted with four attendees of the first two training courses and then integrated into the third course. Participation was voluntary and a total of 30 attendees participated in the third field trip. Visiting different medical institutions (hospital, medical center, a group practice and a single handed practice) gives attendees new perspectives on the professional possibilities associated with rural practice. The first survey showed that 80% of the attendees found it (very) probable that they will later practice in a rural area. After the field trip, 89% reported feeling this way. A total of 96% of attendees were (very) satisfied with the excursion and gave it a rating of 1.3 on the traditional German academic grading scale. Eighty-nine percent of the attendees stated that their attitude toward rural areas had been changed for the positive by the field trip.

##### 3.4.4. Process evaluation

In order to take levels 3 (behavior) and 4 (results) of Kirkpatrick’s model into consideration, the attendees were contacted by telephone three to nine months after completing the training course and asked about their ensuing professional experiences. A total of 34 former attendees have taken part in this process evaluation. Nine participants have found new employment after the training course, and eight have passed the exam necessary for formal professional recognition. A large proportion (29) is still waiting to take the exam. Despite the limit of six months laid down by law, some third-country physicians were still waiting up to 18 months for a date to sit for the exam. The vast majority of them continued to prepare for the exam using the online learning program.

## 4. Discussion and Outlook

This paper presents the approach taken to develop and design a post-licensure training course to aid in qualifying third-country physicians. The aim was to create a program to prepare doctors to practice medicine in Germany, regardless of their desired field of specialty, and to introduce them to the opportunities offered by rural practice. To date, a total of 52 physicians from non-EU member states have attended this training course. Participants were on average 35 years old; 75% of them were male.

### Self-assessment

Surprising were the self-assessments regarding the items “I view it as my responsibility to consider all resources available in the healthcare system when making medical decisions” and “I believe I have developed a good understanding of specific decision-making processes (e.g. ‘watchful waiting’ in general practice),” since a certain level of basic knowledge of the German healthcare system and general medicine must be present to make these claims. However, a need to cover precisely these two areas was indicated in the interviews.

If the results of the self-assessments are compared with the results of an online survey of 95 physicians undergoing post-graduate training [15], it is noticeable that all items, and in particular the items “I feel confident when evaluating scientific studies/publications” and “I believe I have the business skills necessary for practicing medicine,” were rated higher by the attendees in our training course than by the physicians already undergoing post-graduate training in Germany. A total of 56% of the physicians in our courses responded to the question about confidence in evaluating scientific studies with the response categories 1 and 2, while only 27% of the physicians in post-graduate training agreed with this statement. Seventy-three percent of the third-country physicians are of the opinion that they have the business acumen necessary to practice as a general practitioner, while only 56% of the physicians in post-graduate training agreed that they did. The reasons for this could vary. For instance, a lack of knowledge about which business skills are relevant to running a medical practice could lead to an incorrect assessment or overestimation.

Leaving one’s native country may require a greater willingness to take risks, which is perhaps also reflected in the willingness to establish a medical practice. International studies suggest the existence of gender-specific differences. Men appear to assess themselves more optimistically than women in different areas of medicine, including their own medical skills and expertise [19], [20]. Since 75% of course attendees were male, this could provide a further explanation for the results. Further testing of these hypotheses appears worthwhile.

#### Modifications

The project team used the evaluation results and participant feedback to further develop and refine the training course each time it was held again. For instance, the suggestion made during the first course to use medical textbooks more often was implemented in the second course. More practical exercises (e.g. inserting a peripheral venous catheter) and examination techniques were also included starting with the second course. Although the time available for topics pertaining to pharmacology was constantly increased, the evaluations still indicated a greater need.

Nevertheless, the positive evaluations of the course increased each time the course was held again, giving the overall impression that each course was rated more positively than the one before it.

#### Working in a rural area

The intention of this project is to prepare third-country physicians for the practice of medicine in Germany. Particular focus is placed on the possibilities offered by rural practice. In the literature there are other examples of efforts to improve attitudes toward work in rural regions [17], [21], [22]. These mostly involve establishing contact with rural areas. Using interactive measures, attendees were given an impression of a (supposedly) rural area in Schleswig-Holstein and its infrastructure, along with information on the advantages of working in this setting. The course content pertaining to the rural setting resulted in an improvement of individual attitudes. If these results are compared with those of other interventions to introduce physicians to the idea of rural practice [17], it can be seen that the physicians in our sample already had a positive attitude toward working in a rural setting. This positive attitude was reinforced even more by the field trip.

#### Limitations

A selection bias in regard to the interviewees initially surveyed to assess needs cannot be ruled out, the more so because only men could be recruited to represent examiners. Furthermore, there was a basic pre-requisite for participation was German language proficiency at the B2 level, though it cannot be definitively ascertained if all information and evaluation questions were understood equally well by each third-country physician.

The evaluation results presented here were gathered on the basis of a relatively small sample and the process evaluation involving the former attendees is not yet complete. As a consequence, the results here cannot be applied universally.

#### Outlook

The curriculum can be downloaded free of charge from the download-website of the Institute for General Practice (I*nstitut für Allgemeinmedizin*) in Lübeck making it available for use in other German states. Tuition for such a training course can be estimated at around 1,000 € per participant (depending on location).

In order to conduct the evaluation in a way that includes levels 3 and 4 of Kirkpatrick’s model, the former course attendees are monitored for one year after completing the course. The existence of a backlog in the assignment of exam dates should lead to a broader discussion about whether this exam could become part of the M3 state medical exams administered at the universities.

## Funding

The program *Integration durch Qualifizierung* IQ in Schleswig-Holstein, of which this project has been a part, has received funding from the German Federal Ministry of Labour and Social Affairs and the European Social Fund (2015000019-25).

## Acknowledgements

The authors wish to thank the IQ network in Schleswig-Holstein, the *Akademie für medizinische Fort- und Weiterbildung Bad Segeberg*, the course instructors and all of the training course attendees.

## Competing interests

The authors declare that they have no competing interests. 

## Supplementary Material

Guideline for interview questions

Perspectives of the examiners and third-country physicians

## Figures and Tables

**Table 1:**
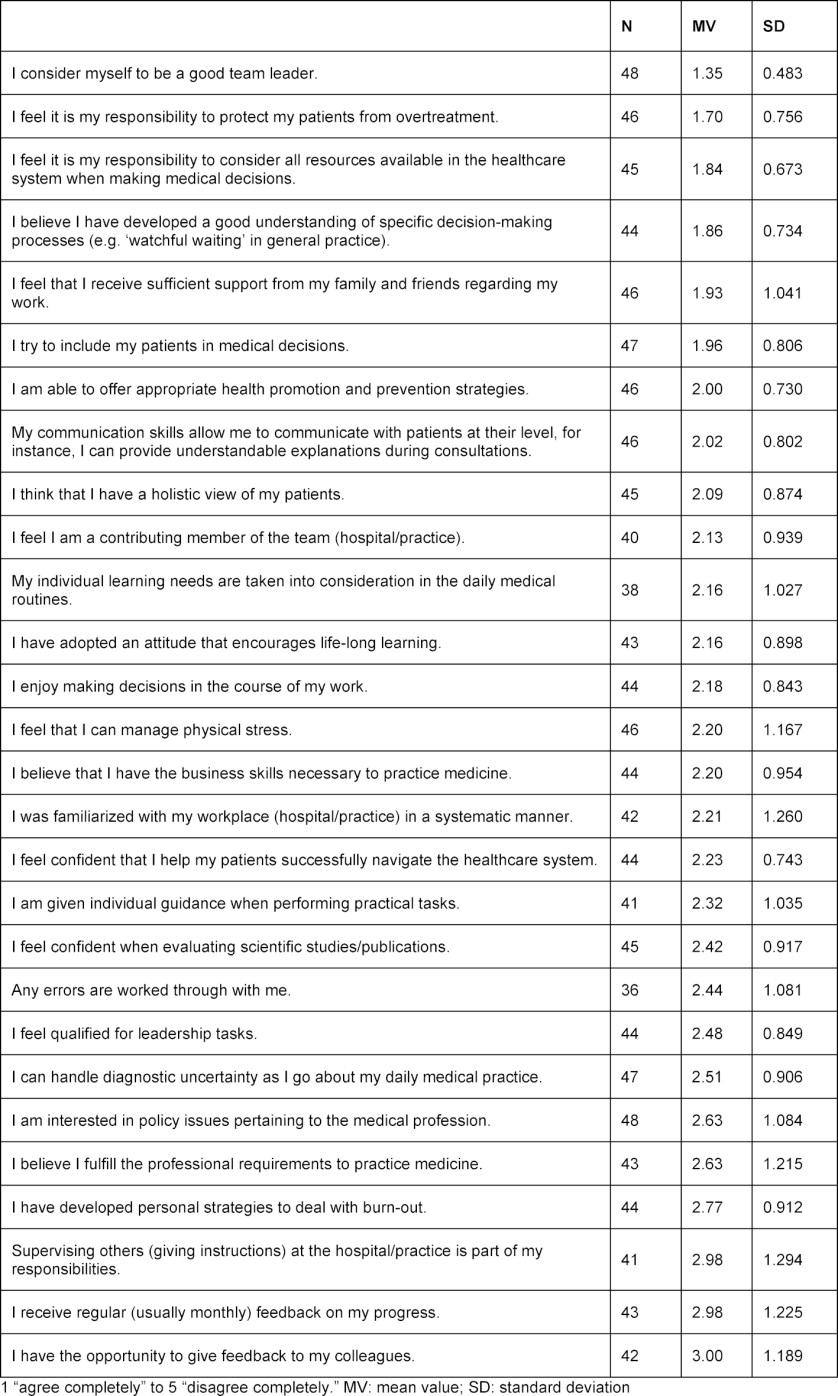
Self-assessment according to the “medical practitioner barometer”

**Table 2:**
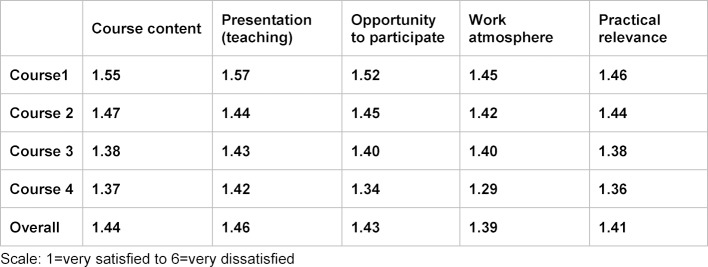
Evaluation of the training program – daily evaluation (n=476)

**Table 3:**
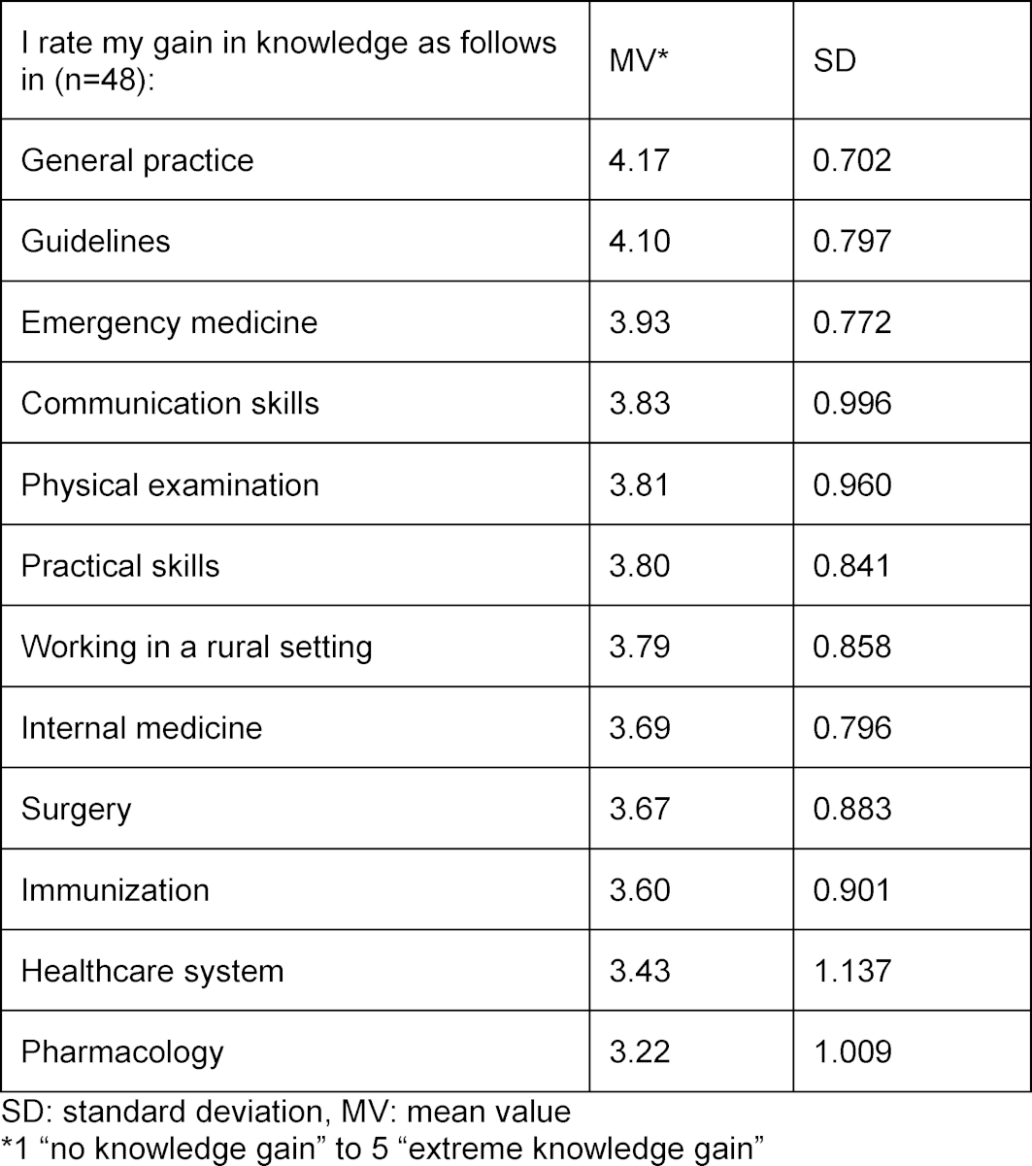
Overall evaluation of perceived knowledge increase
