# Supplementary material for: Strategies for rural areas: The development of and initial experiences with a training course for physicians from third countries to prepare them for medical practice in Germany
Source: GMS J Med Educ. 2019 May 16;36(3):Doc25. doi: 10.3205/zma001233 (PMC6545610; doi:10.3205/zma001233)
Supplement: Guideline for interview questions [file JME-36-3-25-s-001.pdf]

The examiners were asked these main questions:

1. In your experience as an examiner, in which areas have examinees had the greatest deficits?
2. What was the primary reason for an examinee's failure or the exam?
3. Which topics should be covered in a training program for physicians striving to receive professional recognition of a foreign medical degree?
4. What would you like to add that we have not asked about?

The third-country physicians were asked these main questions:

1. In your experience taking exams, in which area did you have the greatest deficits?
2. Which topics should be covered in a training program for physicians striving to receive professional recognition of a foreign medical degree?
3. What would you like to add that we have not asked about?
