# Supplementary material for: Strategies for rural areas: The development of and initial experiences with a training course for physicians from third countries to prepare them for medical practice in Germany
Source: GMS J Med Educ. 2019 May 16;36(3):Doc25. doi: 10.3205/zma001233 (PMC6545610; doi:10.3205/zma001233)
Supplement: Perspectives of the examiners and third-country physicians [file JME-36-3-25-s-002.pdf]

Main category	Code	Example statements	
		**Third-country physicians**	**Examiners**
Deficits	Specific Topics	Yes, perhaps something difficult would be the law or the laws regarding medicine and healthcare. (FG_TN3)<br><br>Yes, all of internal medicine, diabetes, or the heart, myocardial infarction and such general things. (I_1)	Language proficiency remains the greatest deficit. (I_3)<br><br>It is difficult to test topics in general social medicine, by which I also mean occupational safety standards, initiating rehabilitation measures and such things. (I_7)
	Differences between Germany and the country of origin	Pharmacology, I believe. I've noticed that the medications that are used in Germany are not necessarily available in other countries. And, yes, I believe that I do not have good knowledge of this. (FG_TN1)<br><br>Yes, where I come from there is almost no practical training at the university. I believe everyone has heard that medical studies in my country are purely theoretical and there is not much contact with patients. Doctors are hesitant to show what they do or why. (FG_TN5)	
	Differences in Qualifi-cations / Professional experience	I am a specialist for gynecology in my native country [I: Yes.] and for me all of the topics were difficult because for five years all I studied was gynecology. (I_1)	And the second is that there are people from [country] who finished their studies long ago and have accumulated almost no practical clinical experience or they have extremely specialized experience. They are lacking what is considered to be normal basic medical knowledge on the German state medical exams. (I_3)
Reasons for failure	Topics		Yes, you know, if there is a lack of basic knowledge. (I_2)<br><br>People who completed their studies long ago, they are lacking basic understanding. Meaning basic understanding of internal medicine, general practice and surgery. (I_6)<br><br>Clinical knowledge related to clinical examinations. Pharmacology poor ... and, I'll say, in the area of internal medicine and general practice: interpretations of ECGs and X-rays. (I_2)<br><br>In the area of general practice, knowledge that is acquired outside the hospital. (I_4)

			They are relatively poor at connecting the symptoms that are presented to them as a medical case. (I_4)
	Working conditions / Rotation	I work at a <hospital> where I only have surgical cases and I had to learn a little bit for the hospital. But I am always thinking about the exam and I have to learn internal medicine and general practice. And I find that difficult; I can’t do all of that.... (FG_TN2)	This always depends a little on where the examinees worked. If they have been visiting or guest physicians on wards or clinics. If they have been in a surgical department, for instance, then they were pretty much confident with surgery, but then there were deficits in internal medicine. And vice versa. If they were in internal medicine, then surgery was poor. (I_2)
	Exam preparation	At the moment I hardly have time. I have been working...I start at 6:00 am and finish sometimes at 3:00 pm and that is...there’s not enough time. But I have planned my vacation. I will be taking nearly 6 weeks of vacation and I want to use these 6 weeks to study for the exam. But that is not enough. (FG_TN2)<br><br>Yes, I study for two hours every day, and then for about two and half months, but not every day because I also had to work. With children it was difficult....(I_1)	And, I think, personally, that they in part also underestimate the exam or do not properly prepare themselves or are not able to properly prepare themselves. And then they show up with really huge knowledge deficits. (I_6)
Training program	Topics	Emergency surgery, that was the most difficult. (I_1)<br><br>You have to review everything and learn it in German. Yes, but that will go quickly because we have already learned it. (FG_TN5)	A basic course to show how the German healthcare system functions. (I_3)<br><br>Recognizing internistic, surgical emergencies. (I_3)<br><br>This must be included: a structure. That they learn or become familiar with systematic approaches to everyday situations in the German healthcare system. (I_2)<br><br>Case-based learning. (I_4_M)<br><br>They need better knowledge of pharmacology. (I_4)
	Language	First learn German, one must really speak it well, the medical terminology, without an exam on Medical German it is almost impossible to take a medical licensing exam. That is what I have always said, because you really need that before knowledge can be acquired. German language skills in speaking and comprehension come first. (I_1)	The language requirements need to be defined so that those who take the exam have an actual chance of passing it because they have a level of language proficiency that allows them the chance. (I_3)
	Learning aids for exam preparation / Teaching materials	I have to read the "50 internal medicine cases" and there is also "Amboss", which is recommended. And there is also a book, "50 surgical cases" and also internal medicine, "Herold" (FG_TN2)	The use of simulated patients is a good idea for gathering personal experience. (I_3)<br><br>Publications that will appear shortly on a specified topic. Precisely the most common medications, not the most common 100, but

		X-rays should be included and the findings. (I_1_M)	rather the most common 500 and even more, including their side effects and that as part of the curriculum. (I_3_M)
Structures	Structures for recognition of professional qualifications	And the problem here in [German state] is that you cannot take the test if you have no job offer. Without a job offer you cannot take the test and without the test it is impossible to receive a medical license. And when I look for a job I am told by most employers that I need a medical license. It is all connected. (FG_TN2)<br><br>I completed university studies in [German state] and then worked at a large perinatal center in gynecology; I can perform laparoscopy, etc., but then I have to prove that I am a medical doctor? Really? I wanted to return to [country] because I already had a position as senior physician. It was just the theoretical exam—that was rather a problem for me. (I_1)	
	Exam	I heard from my colleagues who have taken the test that the internist is the one in charge and decides if you pass or not. (FG_TN2)	I think it would be best if the physicians would simply take the state medical examination in its entirety, just like our medical students do. And if that were the case, then I would be entirely confident. (I_5)<br><br>But it would mean one more full day of testing: going to the patient bedside as a commission, looking again at clinical examination procedures, clinical tests, and getting a direct impression of the patient case. (I_3_M)
